# Assessment of Circulating microRNAs in Plasma of Lung Cancer Patients

**DOI:** 10.3390/molecules19033038

**Published:** 2014-03-10

**Authors:** Orazio Fortunato, Mattia Boeri, Carla Verri, Davide Conte, Mavis Mensah, Paola Suatoni, Ugo Pastorino, Gabriella Sozzi

**Affiliations:** 1 Tumor Genomics Unit, Department of Experimental Oncology and Molecular Medicine, Fondazione IRCCS Istituto Nazionale dei Tumori, via Venezian 1, Milan 20133, Italy; E-Mails: orazio.fortunato@istitutotumori.mi.it (O.F.); mattia.boeri@istitutotumori.mi.it (M.B.); carla.verri@istitutotumori.mi.it (C.V.); davide.conte@istitutotumori.mi.it (D.C.); mensah.mavis@istitutotumori.mi.it (M.M.); 2 Thoracic Surgery Unit, Fondazione IRCCS Istituto Nazionale dei Tumori, via Venezian 1, Milan 20133, Italy; E-Mails: paola.suatoni@istitutotumori.mi.it (P.S.); ugo.pastorino@istitutotumori.mi.it (U.P.)

**Keywords:** miRNAs, biomarkers, early diagnosis, lung cancer, real-time PCR, haemolysis

## Abstract

Lung cancer is the most common cause of cancer deaths worldwide and numerous ongoing research efforts are directed to identify new strategies for its early detection. The development of non-invasive blood-based biomarkers for cancer detection in its preclinical phases is crucial to improve the outcome of this deadly disease. MicroRNAs (miRNAs) are a new promising class of circulating biomarkers for cancer detection and prognosis definition, but lack of consensus on data normalization methods for circulating miRNAs and the critical issue of haemolysis, has affected the identification of circulating miRNAs with diagnostic potential. We describe here an interesting approach for profiling circulating miRNAs in plasma samples based on the evaluation of reciprocal miRNA levels measured by quantitative Real-Time PCR. By monitoring changes of plasma miRNA-ratios, it is possible to assess the deregulation of tumor-related miRNAs and identify signatures with diagnostic and prognostic value. In addition, to avoid bias due to the release of miRNAs from blood cells, a miRNA-ratios signature distinguishing haemolyzed samples was identified. The method described was validated in plasma samples of lung cancer patients, but given its reproducibility and reliability, could be potentially applied for the identification of diagnostic circulating miRNAs in other diseases.

## 1. Introduction

Lung cancer, for its high incidence and mortality, is the leading cause of cancer-related death in the Western world, with 5-year survival estimates of around 15% for non-small-cell lung cancer (NSCLC) [1]. Despite recent advances in the management of resected lung cancer (*i.e.*, adjuvant chemotherapy) and the use of molecular targeted agents in specific clinical settings, the cure rate remains low due to drug-refractory recurrent disease. 

Prevention, rather than screening, appears the most effective strategy for reducing the burden of lung cancer. The efficacy of lung cancer screening in heavy smokers by low dose spiral-computed tomography (LDCT) remains a controversial issue [2,3]. The largest study performed in the US, the NLST trial [4], showed a 7% reduction in all-cause mortality and −20% lung cancer mortality compared to annual chest X-rays. However the high number of false positive (95%) and the overdiagnosis, recently estimated around the 18.5% [5], increase costs and unnecessary treatments. In addition, the results of three European studies showed no benefit [6,7,8] in overall mortality reduction, underlying the importance of a best selection of high-risk individuals. In this respect, the validation of circulating biomarkers able to identify tumors in a preclinical phase and to track the different aggressiveness of lung tumors, including the early metastatic cancers or even the small lesions with aggressive potential is of paramount importance. 

MicroRNAs (miRNAs) are a large family of small non-coding RNAs, 19–24 nt-long, evolutionarily conserved and tissue specific, that regulate gene expression by causing a block of translation or messenger RNA (mRNA) degradation [9,10]. Such regulation occurs through their binding to partially complementary sequences usually located at the 3'- untranslated region (UTR) of target mRNAs [11,12,13]. They play a critical role in development and differentiation processes of tissues and organs and are aberrantly expressed in different kinds of cancer [14,15]. 

Over the last few years several studies have shown that miRNAs can also be detected within body fluids such as plasma [16], serum [17], sputum [18], saliva [19], urine [20], and milk [21]. Circulating miRNAs have been found packaged in exosomes [22], microvesicles (MVs) [23], or bound to specific proteins such as Ago-2 [24]. Once in the extracellular space, miRNAs could be taken up by other cells (cell-to-cell communication), degraded by RNases, or excreted [25]. Although the mechanism of secretion and incorporation of miRNAs has not been fully clarified, circulating miRNAs may play a pivotal and general role as signaling molecules in physiological and pathological events [26]. 

Notably, circulating miRNAs levels could correlate with cancer progression, therapeutic response, and patient survival, suggesting that they could also be used as non-invasive biomarkers [17,27,28]. In fact miRNAs remain rather intact and stable in the circulation and are also conserved across species, allowing the use of animal models of disease. In addition, miRNAs levels are measurable with simple assays such as RT-qPCR that allows detection of low abundance miRNAs [29,30]. 

The high potential of the circulating miRNAs as molecular marker of disease is diminished by a lack of consensus regarding an optimal method of normalization of miRNA data in plasma samples. The use of small-nucleolar RNAs (snoRNAs), such as RNU6B, have been used to normalise data in some initial studies [31,32], but a few years later it was reported that they vary according to particular diseases and tumour prognosis [33,34,35]. Many authors considered the use of a reference miRNA, such as mir-16, that was later shown to be deregulated in plasma samples of cancer patients [36,37,38,39]. 

The increasing interest in circulating miRNAs as cancer biomarkers has raised the issue regarding the influence of haemolysis on the measurements of miRNAs levels in serum/plasma and the reliability of these data. Pre-analytical and analytical tests able to also identify a minimal haemolysis of samples could be relevant in studies based on circulating miRNAs [40,41].

**Scheme 1:**
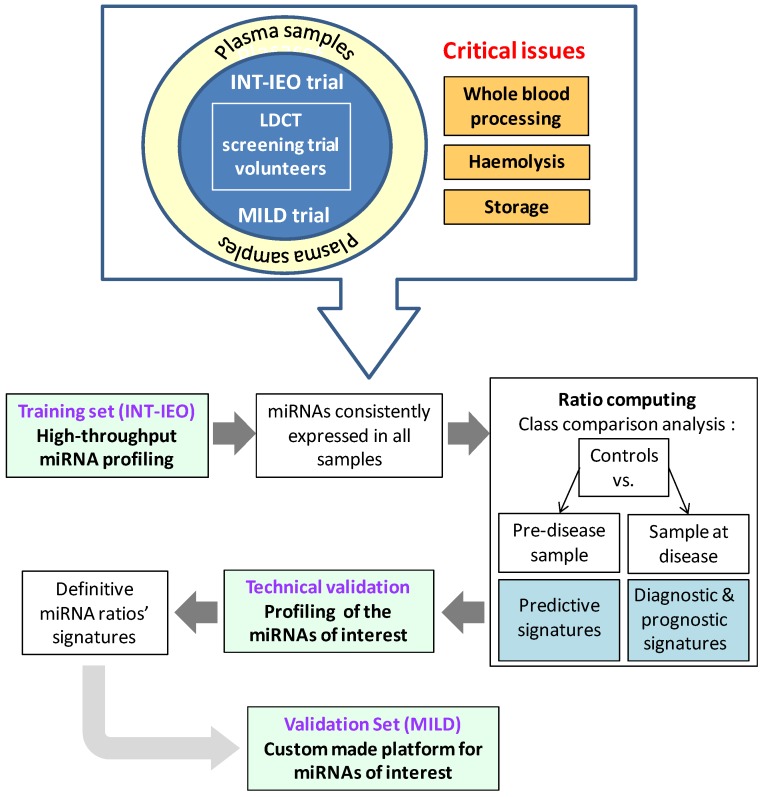
Workflow for circulating miRNA profiling.

In this work, we propose a methodological workflow (shown in Scheme 1) for profiling circulating miRNAs in plasma samples of lung cancer patients that led us to identify miRNA candidates with a potential diagnostic/prognostic role. We employed a standardized method using RT-qPCR microfluidic cards [39] and an innovative data analysis tool for circulating miRNAs that allowed us to circumvent the normalization issue and to consider the impact of haemolysis on plasma samples. Given the high reproducibility and reliability of this procedure, we believe that it could be also used for the development of miRNA-based circulating biomarkers in other cancer diseases.

## 2. Results and Discussion

### 2.1. Whole Blood Sample Processing

**Figure 1:**
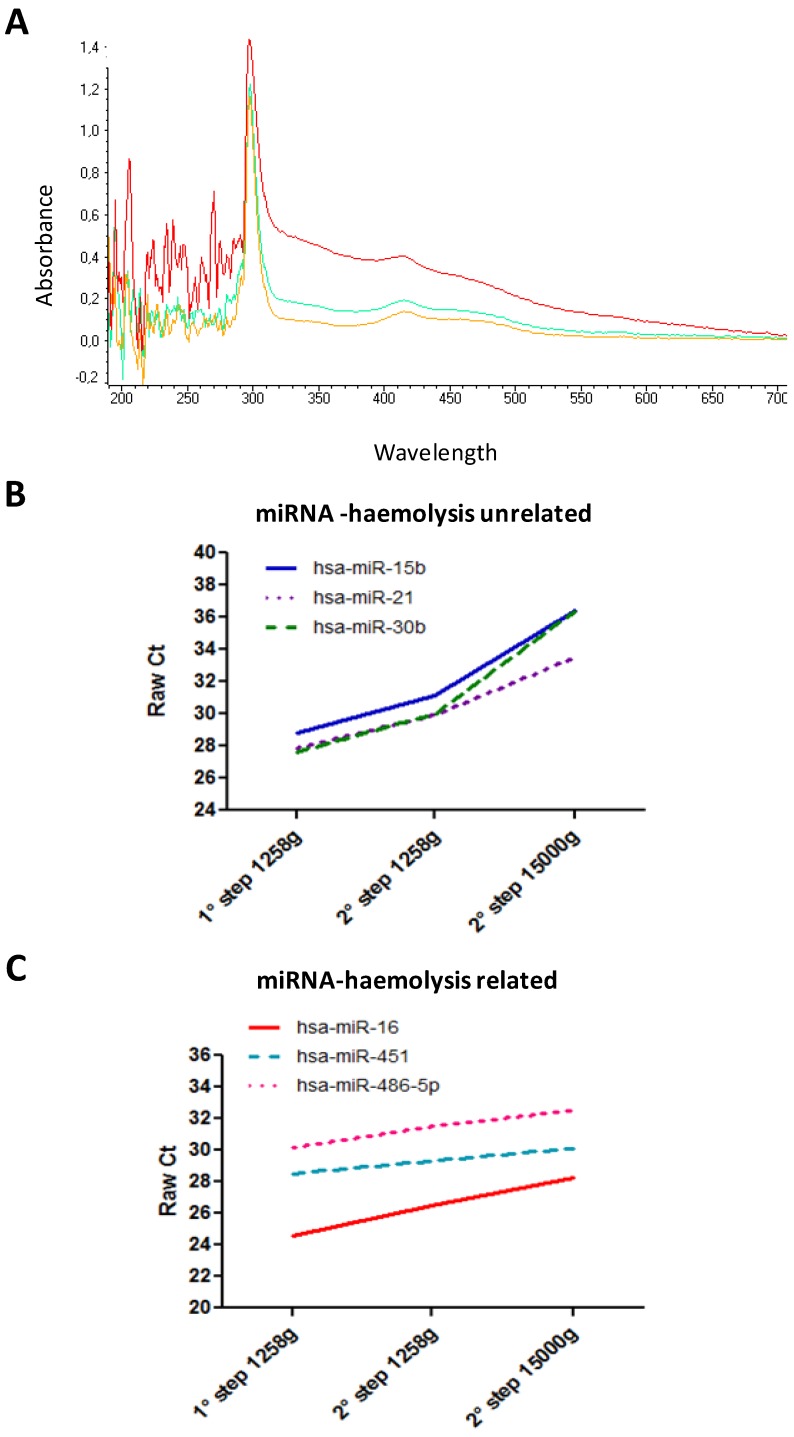
Spectrophotometric and molecular characterization of plasma samples obtained with different protocols of whole blood processing. (**A**) Spectrophotometric profile of samples after the first step of centrifugation at 1,258 g (red line) and after the second step of centrifugation at 1,258 g (blue line) or at 15,000 g (yellow line). (**B**) Expression level (raw Ct data) of haemolysis-unrelated miRNAs and (**C**) of haemolysis-related miRNAs.

During blood samples collection, sudden movements and thermal shocks should be avoided in order to minimize blood cell lysis (haemolysis). Samples should not be stored at 4 °C after blood withdrawal and it is recommended to separate plasma within the first 2 h from blood collection [42]. Centrifugation twice at 1,258 g for 10 min allows obtaining good quality plasma without eliminating plasma components such as lipoproteic complex, microvesicles or exosomes that are known to carry relevant miRNAs. In fact, when we increased the centrifugation speed in the second round (*i.e.*, 15,000g), in order to ameliorate the purity of plasma sample, we observed a change in the spectrophotometric profile of these plasma samples (Figure 1A). Moreover, to evaluate if the expression levels of miRNAs were changed, we assessed the miRNAs levels of three circulating haemolysis-related miRNAs (mir-451; mir-486-5p; mir-16) [40] and three haemolysis-unrelated circulating miRNAs (mir-15b; mir-21; mir-30b,) in haemolyzed plasma samples from four individuals that we have processed following two different protocols: (A) A first centrifugation step at 1,258 g for 10 min, followed by a second centrifugation step at 1,258 g for 10 min (our standard protocol) and (B), a first centrifugation step at 1,258 g for 10 min followed by a second centrifugation step at 15,000 g for 10 min. We analyzed miRNAs expression by Real Time PCR according to the Multiplex RT-PCR Protocol for TaqMan MicroRNA assay (Life Technologies, Carlsbad, CA, USA). No proportional reduction was observed in protocol (B) for the six miRNAs, since the levels of haemolysis-related miRNAs decreased to a less and not comparable extent to other miRNAs (Figure 1B,C). This finding suggest that miRNAs not released in physiological process, as during cell lysis, have a different physical state than miRNAs physiologically released and protected by lipoproteic complex or microvesicles. Thus, our plasma separation protocol (A) allows one to avoid a miRNA selection based on their physical status.

### 2.2. MiRNAs Profiling

The amount of total RNA extracted from 200 μL of plasma and eluted in 50 μL of elution buffer (Life Technologies) was measured with a spectrophotometer, and showed final concentrations less than 10 ng/μL with a 260 nm/280 nm ratio of less than 1.2. Despite this poor quality of the total RNA, the miRNAs fraction is well known to be stable and intact when extracted from plasma samples [43]*.* Given the unfeasibility of exactly quantifying the amount of the miRNAs fraction in the total RNA content, a fixed volume of 3 μL of eluted RNA was used for miRNA profiling. A first set of 67 plasma samples from subjects enrolled in a lung cancer spiral CT-screening trial was analyzed with a high-throughput approach, such as TaqMan Array Human MicroRNA microfluidic Cards A (Life Technologies). One hundred thirteen of the 384 miRNAs spotted on each card, were measurable in plasma samples and 100 of them were strongly reproducible between biological duplicates.

Starting from these 100 circulating miRNAs, we performed analysis comparing plasma samples from lung cancer patients and disease-free individuals and the miRNAs found differentially expressed were further validated following the Multiplex RT Protocol for TaqMan MicroRNA assay (Life Technologies).

### 2.3. MiRNAs Ratios Computing

**Figure 2:**
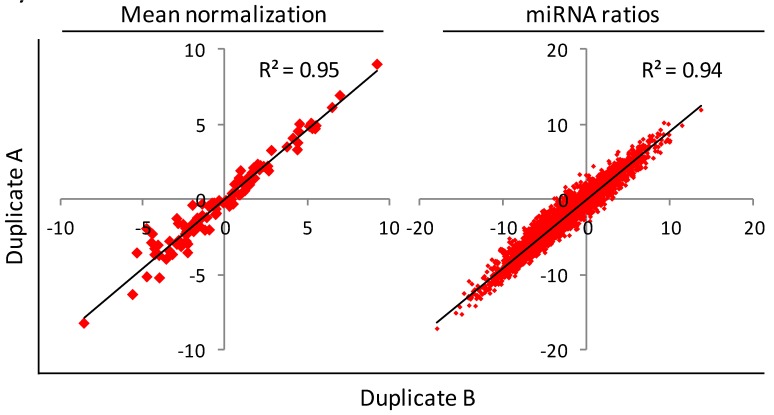
Scatter plots showing the correlation using the mean normalization method and the miRNAs ratios tool, between two biological replicates spotted on microfluidic cards analyzed for the reproducible 100 miRNAs and the respective 4,950 ratios.

To analyze large amounts of data such as those obtained from microfluidic cards, there is a general agreement in using normalization on the mean expression value of all miRNAs [44]. However, when a few circulating miRNAs have to be analyzed, as in the technical validation, no suitable housekeeping miRNAs for plasma samples are known. Thus, to bypass the normalization issue, miRNA values were analyzed looking at the reciprocal ratios among the 100 circulating miRNAs. In this way all the ΔCts between each miRNA and the other 99 were considered and the resulting 4,950 features were used for the subsequent classification of plasma samples. When using the miRNA ratios tool for RT-qPCR data, the same signal threshold for all miRNAs in all samples has to be fixed to obtain comparable Ct values. To validate the robustness of the miRNA ratios method, correlation between biological duplicates was analyzed by either normalization on mean expression value or using the miRNA ratios tool, showing correlation coefficients (R2) of 95% and 94%, respectively (Figure 2). Moreover, the miRNAs most significantly deregulated in the class comparison normalizing on the mean, were those most redundant in the analysis using ratios (Table 1).

Using the miRNA ratios tool we were able to identify predictive, diagnostic and prognostic signatures composed by a total of 24 miRNAs differentially expressed in plasma of lung cancer patients and disease-free individuals collected during two low dose computed tomography (LDCT) screening trials performed in our Institute: a completed pilot observational study (INT-IEO) and an ongoing randomized trial (MILD) [39]. In order to further apply the test in clinical practice, we validated its performance using custom made microfluidic cards, containing only the 24 selected miRNAs, in an extended series of 69 patients and 870 disease-free individuals belonging to the MILD screening trial [45]. In addition, by analyzing such a large number of samples, we were also able to better define critical issues affecting the analysis on circulating miRNA biomarkers. 

**Table 1:** Comparison between the results obtained normalizing on the mean expression value or using the miRNA ratios tool.

	Mean Normalization		miRNA Ratios Tool	
miRNA	*p*-value *	Mean patients/mean controls (log2)	No. ratios with *p*-value < 0.01 ^†^	Direction in patients *vs.* controls
mir-660	3.5E-05	−1.8	56 ^†^	All down
mir-142-3p	3.0E-04	−0.9	34	All down
mir-197	4.9E-03	0.9	35	All up
mir-24	0.4	0.16	13	4 Up & 9 Down

* Two-tailed Fisher’s Exact Test; ^†^ On a total of 99 ratios for each miRNA.

### 2.4. Effect of Storage Time on Plasma miRNAs

**Figure 3:**
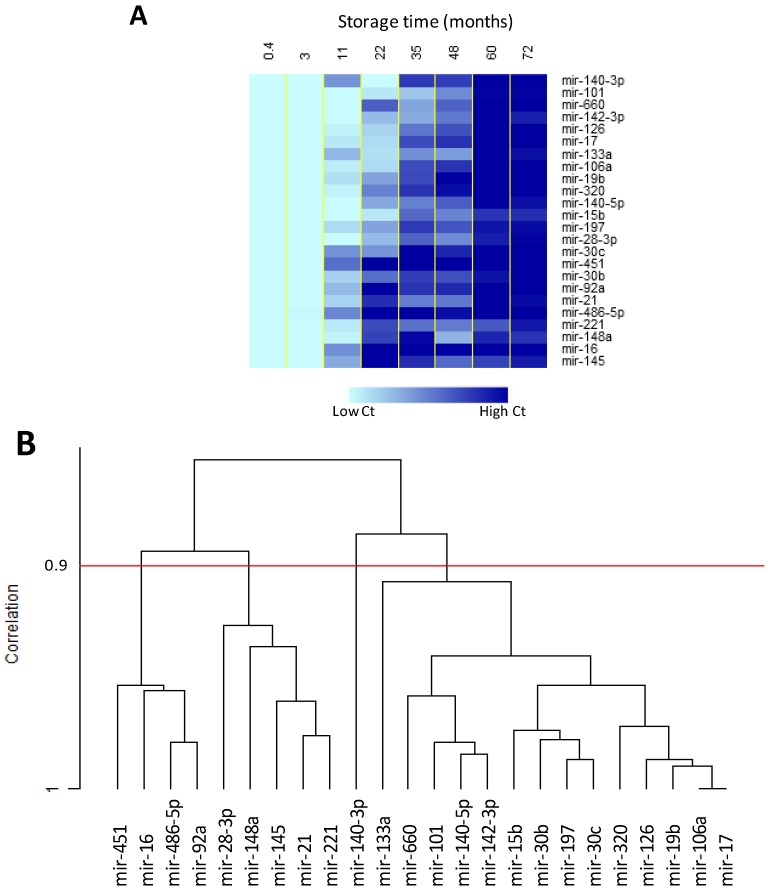
miRNA degradation according to storage time reported as (**A**) heat map of the time course analysis, and (**B**) dendogram using centred correlation and average linkage for miRNAs clustering. Analysis performed with BRB ArrayTools.

Plasma samples from 64 disease-free individuals were profiled to specifically assess if storage time could affect miRNAs levels in frozen plasma samples. The averages of raw Ct data among groups of eight samples at eight longitudinal storage time-points (from 0 to 72 months) were used to cluster the 24 miRNAs. As expected, all the miRNAs were degraded in plasma samples according to storage times (Figure 3A) [39]. 

However, not all the miRNAs had the same degradation profile and in particular using a correlation threshold of 0.9, miRNAs clustered in four groups according to degradation time (Figure 3B). The first cluster was composed of the four miRNAs (miR-16, 451, 486-5p and 92a) reaching the higher Ct in the shortest time (11 months); the second cluster was composed of miR-28-3p, miR-145, miR-148a, miR-21 and miR-221 (22 months); whereas miR-140-3p and 14 other miRNAs were grouped after 35 months. The four miRNAs degrading faster were exactly the same four miRNAs previously found to be freely released in the circulation system by haemolysis [40], thus suggesting that the differences in degradation time could be related to the physical status of circulating miRNAs: free, bound to proteins or included in exosomes or microvesicles.

### 2.5. Evaluation of Haemolysis in Plasma Samples

#### 2.5.1. Pre-Analytical Quality Control

**Figure 4:**
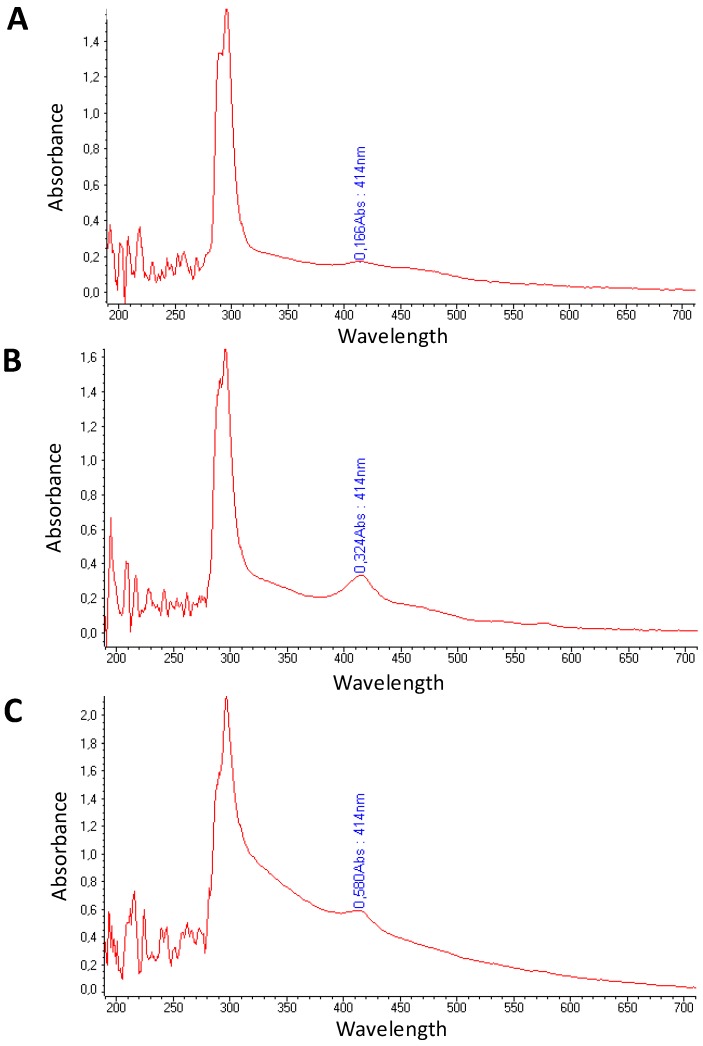
Spectrophotometric measures of haemoglobin (414 nm) of (**A**) a normal, (**B**) a haemolysed and (**C**) a lipemic plasma sample.

A critical issue, recently emerged, for the analysis of circulating miRNA as biomarkers in plasma/serum samples, was the contamination of these samples by miRNAs released by blood cell haemolysis [40,41,46,47]. In order to check our samples for haemolysis in a pre-analytical phase, before the total RNA extraction step and miRNAs profiling, we measured the absorbance of plasma samples at 414 nm, 541 nm and 576 nm wavelengths (Figure 4) to identify the presence and the amount of free hemoglobin in the samples [41,48]. Since we observed that the absolute value of the peak at 414 nm is not fully representative of the level of haemolysis due to the lipid content of plasma sample that interferes with spectrophotometric measurements (Figure 4C), we introduced a further absorbance measurement at 375 nm, where the wavelength spectrum is more affected by the lipid content of the samples, to “normalize” the 414 nm peak value. 

#### 2.5.2. Haemolysis-Related miRNA Signature

To get insight into the haemolysis issue, 24 plasma samples haemolyzed at visual inspection (color range) and 30 plasma samples not visually haemolyzed, were profiled using custom made microfluidic cards containing the 24 selected miRNAs previously identified. The four haemolysis-related miRNAs: mir-16, mir-451, mir-486-5p and mir-92a [40], together with mir-101 and mir-140-3p (not previously reported), resulted, using restrictive cut-off (Δ > 2Ct), to be significantly up-regulated (*p* < 0.001) in the 24 haemolyzed samples (Table 2).

**Table 2:** Raw Ct values of miRNAs in haemolysed and not haemolysed samples.

	24 Haemolysed Plasma Samples *		30 Not Haemolysed Plasma Samples *			
	Ct average	s.d.	Ct average	s.d.	*p*-value	ΔCt
miR-101	28.8	1.8	31.0	1.4	0.000	2.2
miR-106a	19.9	1.6	21.4	1.3	0.014	1.5
miR-126	21.7	1.5	21.9	1.4	0.484	0.3
miR-133a	30.8	2.4	30.8	1.6	0.964	0.0
miR-140-3p	28.9	1.9	32.1	1.6	0.000	3.1
miR-140-5p	25.8	1.6	26.4	1.2	0.177	0.5
miR-142-3p	22.3	1.8	22.1	1.5	0.643	−0.2
miR-145	26.6	1.8	27.1	1.5	0.258	0.5
miR-148a	28.6	1.5	29.8	1.6	0.012	1.1
miR-15b	25.0	1.6	25.9	1.4	0.034	0.9
miR-16	18.3	2.2	21.6	1.2	0.000	3.3
miR-17	20.0	1.6	21.5	1.3	0.010	1.5
miR-197	26.2	1.2	27.0	1.4	0.035	0.8
miR-19b	20.4	1.8	22.2	1.3	0.007	1.9
miR-21	24.9	1.9	25.5	1.2	0.172	0.6
miR-221	24.8	1.9	24.7	1.5	0.904	−0.1
miR-28-3p	26.8	1.4	27.1	1.4	0.352	0.4
miR-30b	22.8	1.6	23.1	1.5	0.461	0.3
miR-30c	24.3	1.6	25.1	1.5	0.104	0.7
miR-320	22.6	1.4	23.8	1.3	0.018	1.2
miR-451	20.0	2.5	23.9	1.4	0.000	3.9
miR-486-5p	20.6	2.2	24.0	1.3	0.000	3.3
miR-660	27.8	2.1	29.6	1.3	0.009	1.8
miR-92a	22.8	1.5	24.7	1.2	0.000	2.0

**Figure 5:**
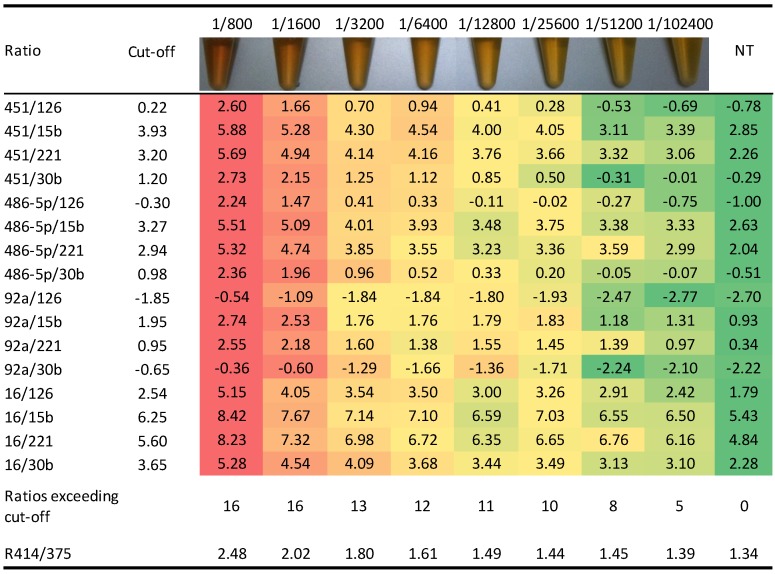
Plasma ratios (log_2_) for *in vitro* haemolysis evaluation.

In order to use miRNAs ratios to identify haemolyzed samples, serially diluted lysed red blood cells (RBCs) were introduced in non-haemolyzed plasma samples as an artificial working model. Four non-haemolysis related miRNAs were identified (mir-126, mir-15b, mir-221 and mir-30b), whose reciprocal ratios with the four haemolysis-related miRNAs better correlated with serial RBCs dilutions (Figure 5). A cut-off for each of the 16 ratios composing the haemolysis signature was then fixed as the value exceeding two standard deviations from the average of the values among the 30 non-haemolyzed samples. When at least eight out of 16 miRNAs ratios (50%) exceeded their respective cut-offs(corresponding to a spectrophotometer ratio 414/375 nm > 1.4), the miRNA signatured showed 88% sensitivity and 93% specificity to classify the 24 haemolyzed and 30 non-haemolyzed (visual inspection) plasma samples. 

In conclusion we have established an absorbance ratio (414/375 nm) test and a miRNAs ratios signature able to efficiently classify haemolysis in plasma samples. These assays can be used in combination since absorbance ratio can be analyzed in a pre-analytical phase in a cost-effective manner, whereas haemolysis-related miRNA signature can be used as quality control in a post-analytical phase.

NT = not treated.

## 3. Experimental

### 3.1. Study Population and Plasma Sample Collection

Our recent studies were focused on miRNAs as biomarkers for the identification of lung cancer at its earlier stages or even before the tumor is fully developed. Since the samples were collected all along the two LDCT screening trials, we had the opportunity to study miRNAs expression profiles in plasma samples longitudinally collected either at the time of cancer diagnosis and also before disease detection. 

Whole blood samples (5–10 mL) were collected as first blood withdrawal in BD Vacutainer tubes with spray-coated K_2_EDTA (BD-Becton, Dickinson and Company, Plymouth, UK) and stored at room temperature. Within 2 h, plasma was separated by a first centrifugation step at 1,258 g at 4 °C for 10 min. We carefully transferred the supernatant containing plasma into a 15 mL tube avoiding collecting the fraction closest to the lymphocytic ring. Plasma was then centrifuged a second time at 1,258 g at 4 °C for 10 min. Finally we harvested plasma aliquots, avoiding collection of the plasma fraction in the bottom of the tube near the debris pellet and stored them at −80 °C, with the exception of one aliquot that was used for the evaluation of haemolysis.

### 3.2. Evaluation of Haemolysis by Spectrophotometry

Immediately after the separation step, plasma sample was diluted (1:10) in PBS in a cuvette for spectrophotometer (10 mm path length), and mixed to homogenize the diluted sample. The absorbance at 375 nm, 414 nm, 541 nm and 576 nm was measured using a NanoDrop 2000c instrument (Thermo Scientific, Wilmington, DE, US) in UV-Vis configuration, with pre-selected “baseline correction” setting (750 nm) and a path length of 10 mm. The measurement of 1 mL of PBS was performed as a blank.

### 3.3. RNA Extraction and miRNA Profiling

Starting from 200 μL of plasma, total RNA extraction was performed according to the protocol for biological fluids of the mirVana PARIS Kit (Life Technologies). To obtain more concentrated total RNA, samples were eluted in 50 μL of Elution Solution pre-heated at 95 °C, instead of 100 μL and then stored at −80°C.

*High-throughput screening*: Plasma samples of the training set were profiled using TaqMan Array Human MicroRNA microfluidic Cards A (Life Technologies) containing 384 miRNAs assays following the standard Megaplex Pools protocol. Since we started from a small amount of total RNA, a pre-amplification step of 12 cycles was required to increase, with an unbiased approach, the quantity of specific cDNA targets of all 384 miRNAs. 

*Single assay validation*: The validation of data obtained in the training set was performed in an independent set of samples with single assays following the Multiplex RT Protocol for the TaqMan MicroRNA assay (Life Technologies). Differently from the Megaplex Pools protocol, the Multiplex RT Protocol started from 4 μL of total RNA and did not require any pre-amplification step, thus the sensitivity of the assay was reduced.

*Custom made microfluidic cards*: Custom made TaqMan MicroRNA microfluidic cards containing the 24 selected miRNAs in duplicate was conceived to analyze a large number of samples and to standardize the protocol. Eight samples were analyzed simultaneously in each card, following the manufacturing protocol for Creating Custom RT and Pre-amplification Pools with TaqMan MicroRNA Assays (Life Technologies). Briefly, 3 μL of total RNA were converted into cDNA by performing a reverse transcription (RT) reaction for all 24 miRNAs using the TaqMan MicroRNA Reverse Transcription Kit and a Custom TaqMan RT Primer Pool with the 24 miRNAs of interest. In this protocol the pre-amplification of cDNA was required, thus 2.5 μL of each RT product were pre-amplified using a Custom TaqMan PreAmp primer pool and 1.13 μL of diluted pre-amplified cDNA was used for real-time PCR reaction. Custom miRNA microfluidic cards were run using the ViiA7 Real-Time PCR System modifying the thermal cycling parameters as follows: 10 min at 94.5 °C followed by 40 cycles at 97 °C for 30 s and at 59.7 °C for 60 s.

### 3.4. Data Extrapolation and Ratios Generation

Raw Ct values were obtained using the ViiA7 RUO software, setting a fixed threshold of 0.15 and an automatic baseline. Spots with bad amplification curves or with awful passive reference signals were excluded from the analysis. The average of each miRNA-duplicate has been considered to calculate −ΔCts between each miRNAs and the others, corresponding to the log2 values of the miRNA ratios.

### 3.5. Storage Time Evaluation

Plasma samples were profiled at serial storage time-points (ranging from 0 to 72 months) using custom microfluidic cards. The averages of raw Ct data among groups were used to run time course analysis and to cluster the 24 miRNAs using centred correlation and average linkage with BRB ArrayTools. A correlation threshold of 0.9 was used to define the clusters.

## 4. Conclusions

The challenge for the next decade will be to bring biomarkers into the clinic in ways that are efficient and practical. Circulating miRNA expression profiles, not single or couples of miRNAs, that constitute the fingerprint of physiological or pathological conditions, have the potential to overcome the limitations of single blood-based biomarkers in clinical use so far, and may strongly impact early cancer diagnosis. Here we report an experimental design that allows one to identify a panel of circulating miRNAs deregulated in subjects who developed lung cancer during LDCT screening trials compared to disease-free heavy smokers. Of interest, the analytical approach described, based on the reciprocal levels of miRNAs (miRNA ratios), is appealing since it allows one to bypass the issue of data normalization using controversial “housekeeping” miRNAs and proved to be more informative on the disease status rather than absolute levels of individual miRNAs.

Our preliminary results [39] showed that specific miRNAs and miRNA ratios were also able to discriminate patient plasma samples when the LDCT was still negative. We reasoned that such circulating biomarkers would not derive exclusively from tumor tissue, but rather from a lung environment that may favor tumor development. Based on these considerations, we preferred a high-throughput miRNA screening rather than a tumor-driven approach. In addition, the multi-step validation process, first on a small validation set using a different methodological approach such as the single assay quantitative Real Time PCR, and afterwards in a enlarged validation set using custom miRNA microfluidic cards, confirmed the great potential of miRNA biomarkers, as well as their reproducibility and reliability. At the moment, functional studies using proper *in vitro* and *in vivo* models are ongoing in order to better understand if these circulating miRNAs have a functional role in lung cancer.

The technical approach proposed is based on molecular assays with high performance and sensitivity. For this reason, when this strategy has to be applied on blood-derived samples, such as plasma and serum, pre-analytical and analytical aspects should be carefully evaluated. The haemolysis of blood samples is the first issue that should be considered, given that low-level haemolysis is a frequently occurring event during plasma collection that can influence the measurement of any candidate miRNA biomarker also expressed by red blood cells. Our study identified a miRNA signature able to detect slight levels of haemolysis, that avoids having results affected by unspecific release of miRNAs. 

A further concern is the effect of storage on miRNA levels. By studying a large series of samples collected during a LDCT screening trial, we found that the miRNA expression values in plasma samples collected in different years of the screening program have different expression ranges. Thus, we suggest that for class comparison studies case and controls series with a similar period of storage have to be compared and in prospective studies miRNA ratio cut-offs must be checked on freshly collected plasma samples.

Despite these considerations, we believe that circulating miRNAs have great potential as molecular markers in cancer diagnosis and prognosis, since deregulation of these molecules is detectable up to two years before the diagnosis with imaging tools is possible. Moreover, the analytical approach proposed and the use of a simply and conventional technique such as the RT-PCR, improve the feasibility of using miRNAs as circulating biomarkers. We have reported here a validated method for biomarker discovery in plasma samples of lung cancer patients and heavy smoker controls, that given its reproducibility and robustness, could also be potentially applied for the identification of diagnostic circulating miRNAs in other diseases.
